# Population Differences in Postural Response Strategy Associated with Exposure to a Novel Continuous Perturbation Stimuli: Would Dancers Have Better Balance on a Boat?

**DOI:** 10.1371/journal.pone.0165735

**Published:** 2016-11-02

**Authors:** Carolyn A. Duncan, Tony G. J. Ingram, Avril Mansfield, Jeannette M. Byrne, William E. McIlroy

**Affiliations:** 1 Toronto Rehabilitation Institute, University Health Network, Toronto, ON, Canada; 2 Department of Kinesiology, University of Waterloo, Waterloo, ON, Canada; 3 Laboratory for Brain Recovery and Function, Dalhousie University, Halifax, NS, Canada; 4 Department of Physical Therapy, University of Toronto, Toronto, ON, Canada; 5 Evaluative Clinical Sciences, Hurvitz Brain Sciences Research Program, Sunnybrook Research Institute, Toronto, ON, Canada; 6 School of Human Kinetics and Recreation, Memorial University, St. John’s, NL, Canada; University of Ottawa, CANADA

## Abstract

Central or postural set theory suggests that the central nervous system uses short term, trial to trial adaptation associated with repeated exposure to a perturbation in order to improve postural responses and stability. It is not known if longer-term prior experiences requiring challenging balance control carryover as long-term adaptations that influence ability to react in response to novel stimuli. The purpose of this study was to determine if individuals who had long-term exposure to balance instability, such as those who train on specific skills that demand balance control, will have improved ability to adapt to complex continuous multidirectional perturbations. Healthy adults from three groups: 1) experienced maritime workers (n = 14), 2) novice individuals with no experience working in maritime environments (n = 12) and 3) individuals with training in dance (n = 13) participated in the study. All participants performed a stationary standing task while being exposed to five 6 degree of freedom motions designed to mimic the motions of a ship at sea. The balance reactions (change-in-support (CS) event occurrences and characteristics) were compared between groups. Results indicate dancers demonstrated significantly fewer CS events than novices during the first trial, but did not perform as well as those with offshore experience. Linear trend analyses revealed that short-term adaptation across all five trials was dependent on the nature of participant experience, with dancers achieving postural stability earlier than novices, but later than those with offshore experience. These results suggest that long term previous experiences also have a significant influence on the neural control of posture and balance in the development of compensatory responses.

## Introduction

Humans’ ability to control upright, bipedal stability presents a complex neuromechanical challenge that requires a sophisticated coordination between ‘reactive’ and ‘predictive’ balance control [1]. Research examining reactive control has found that it requires complex multi-segmental control that must simultaneously meet the challenges of the imposed instability and surrounding environment, while at the same time being executed at remarkable speeds [1, 2]. What is also clear is that optimizing reactive control, and maximizing stability, requires predictive control that is informed by expectation and past experience. Predictive control is evident in task-specific situations where the ability to anticipate instability affords execution of avoidance or optimization of control [3]. Such predictive control is also the foundation for short and long-term skill development where dynamic stability control is essential.

The central nervous system (CNS) uses past experiences and expectations to pre-set descending commands that optimize task-specific balance control either by adopting anticipatory control strategies or by modulating CNS state to influence reactions to perturbations [4]. The CNS expression of the influence of experience is referred to as postural or central set [5]. There are two time frames upon which such adaption can occur: 1) short term (minute to minute or trial to trial changes); and 2) long term (associated with skill learning). Evidence of short term adaptations associated with changes in central set can be found in studies using repeated presentation of discrete postural perturbations. For example, repeated application of perturbations leads to very rapid trial to trial adaptations thought to be associated with explicit or implicit learning of stimulus properties [6, 7,8]. Studies have also revealed anticipatory changes in physiological responses that have been used to infer changes in central set. Evidence of pre-perturbation cortical activity prior to temporally predictable instability serves as indirect evidence of predictive changes in CNS state [9, 10]. In addition, changes in physiological arousal, measured via galvanic skin responses, and reflex excitability that are associated with standing and height are also thought to reflect changes in central set [6, 7, 8, 9, 10, 11]. Collectively these observations reveal evidence of short-term changes in CNS state associated with prior experience and/or expectation.

What is of specific interest in the present study is whether longer-term prior experiences, requiring challenging balance control, carryover as adaptations that influence the ability to react in response to novel stimuli. At the root of this question is whether those who train on specific skills that demand sophisticated balance control will have improved capacity to solve unique task-specific balance challenges. There is evidence of the unique balance control abilities in individuals who have specific skill training such as dancers and gymnasts [12, 13, 14, 15] or those with specialized occupational training (e.g. maritime workers [16]). It is anticipated that learned associations between the characteristics of the challenges to stability (both internal and external perturbations) and the associated balance recovery strategies comprise a skill specific central state. Central state can be conceptualised as the context specific internal model that is currently loaded in an individual’s working memory, which represents a set of stimulus (a balance perturbation in the case of the current study) to response (a particular recovery strategy) associations (central set).

Moving environments, like those experienced by individuals working in maritime industries such as commercial fishing, shipping and offshore petroleum, provide a unique example of a complex and continuous multi-directional threat to stability. In order to maintain balance and perform their required duties, without falling and resultant injury, individuals working in these environments must overcome these threats. Previous research suggests that the postural responses in these moving environments are highly variable and cannot be predicted based on the task or perturbation characteristics in isolation [17, 18]. Repeated exposure to wave-induced moving environments result in rapid changes in postural activity among individuals with no previous experience in these environments [18, 19]. What is of interest in the present study is whether individuals who have developed skill-specific expertise in balance control are able to use this central set to enable them to adapt more quickly when exposed to novel balance perturbations. This line of inquiry is important to the fundamental understanding of the role of previous experiences in neural optimization of postural responses and skill acquisition to a novel stimulus. Therefore, the purpose of this research was to determine how prior experience influences balance responses to a novel continuous wave-like perturbation. It was hypothesized that those with prior experience in balance demanding skills such as dancing would be able to rely on a learned central state that enables them to more rapidly adapt to the novel perturbations imposed by the moving platform. Despite their ability to adapt more quickly than individuals with relatively little pre-existing balance expertise, the balance responses of these individuals would not be as effective as experienced maritime workers whose central state is more specifically adapted to the unique task-specific properties of wave motion.

## Materials & Methods

### Participants

Thirty-nine healthy adults (18 men, 19 women, Table 1) were recruited. Participants were members of one of three groups: 1) experienced maritime workers (Experienced Workers group; n = 14); 2) individuals with training in dance (Dance group; n = 12); and 3) novice individuals with no experience working in maritime environments or dance training (Inexperienced group; n = 13). Anthropometric data was normally distributed with no statistically significant between group differences. To qualify for the study members of the Inexperienced group had to have no experience working in offshore environments, less than two weeks experience in recreational boating activities, and no formal dance or skill training requiring challenging whole body balance control. Experienced Workers had to have a minimum of six months experience working in maritime environments on moving platforms. Individuals in the Dance group must have had a minimum of two years of formal dance training in either classical or street dance genres, and be practicing and/or performing at least two hours per week over the previous six months. Individuals were excluded if they were susceptible to motion sickness; had any medical conditions that would adversely affect balance, or any musculoskeletal injuries or other impairments that would prevent them from safely exercising. Prior to commencing the study all participants were presented with documentation outlining the study and were given the opportunity to ask questions about the research before signing the informed written consent form. This study was approved by the Interdisciplinary Committee on Ethics in Human Research at Memorial University of Newfoundland.

**Table 1:** Participant anthropometrics by group.

Group	Experienced Workers	Dance	Inexperienced
**Age (years)**	30.9 ± 5.3	27.1 ± 4.2	26.8 ± 4.7
**Height (cm)**	174.3 ± 7.0	173.9 ± 8.8	172.8 ± 9.6
**Weight (kg)**	81.5 ± 13.3	73.6 ± 11.8	74.9 ± 13.6
**Experience (years)**	6.2 ± 5.7	5.8 ± 4.6	Not applicable

Note: Data presented as mean ± standard deviation. Between group differences did not reach statistical significance (p > .05) for each measure.

### Procedures

Participants performed a total of five trials. During each trial they stood at the centre of a platform (2 m^2^) that was capable of moving in six degree of freedom. All trials were 5 minutes in durations and were separated by a break of 2–3 minutes. For all trials participants were instructed to move their feet as desired, whenever it was felt necessary to maintain balance. Stance width was self-selected by each participant. All trials were performed with the participants facing the front of the motion platform. All motion trials were performed over a two-hour session.

The platform moved in a manner that replicated ship board motions and consisted of periodic motion in five of the six available degrees. Motion was based on time series data collected during previous research that examined the deck motion of floating vessels of various sizes. These recorded ship motions were reconstructed using linear wave theory [20] (Eqs 1–5). Yaw was not introduced within the motion profiles due to the small amplitudes relative to the other angular motions under typical conditions. A canopy was placed over the motion platform to limit the effect of earth referenced visual cues.

Roll =0.8(6sin(1.050t)+1.25sin(0.11t+0.5)) (1)

Pitch =0.8(2.5sin(1.76t+0.5)+sin(t)−1.5) (2)

Heave=0.1(5sin(1.595t+2)+15sin(1.21t)) (3)

Surge=0.1(7.8sin(0.649t+4.8)+7.8sin(0.825t+3.8)+0.5) (4)

Sway=0.1(18sin(0.583t+5)+9sin(1.122t+5.4)−0.25) (5)

### Data Acquisition and Statistical Analysis

Trials were recorded using a digital video camera at 60 Hz, and centre of pressure was recorded using a 0.5 m x 1 m Tekscan Walkway foot pressure system (Tekscan Inc., South Boston, Massachusetts).

Video and Walkway data were examined to determine when participants used a change-in support (CS) strategy to maintain stability on the platform. A CS strategy was considered to be any instance when the participant stepped from their original position or grabbed the guard rail during the trial. Any stepping motion performed within one second of another was considered to be part of the previous CS [21]. The characteristics of CSs that examined were:

1. Total time spent performing CSs (% Trial)–percentage of trial
2. Steps (# Steps)–total count
3. Multiple step CS (# Mulitstep) event occurrence—stepping events in which one or more steps occurred within one second of another

At the beginning of each trial the video camea was first started. The Tekscan walkway and then motion platform were then started. Tekscan Walkway and motion platform were synchronized at the initiation using a trigger. To facilitate this analysis, the video camera was to the platform and Walkway systems post-collection using visual cues from the platform that indicated platform initiation. The CS initiation times were determined from centre of pressure data while the event characteristics were later determined from video recordings of the trials.

For all CS variables of interest group differences for the first trial only were examined using a one-way analysis of variance (ANOVA) to examine the effects of prior experience on task performance. Where homogeneity of variance was violated Welch’s robust test was used. Group differences for all trials were examined using two-way repeated measures ANOVA with factors of group (Inexperienced, Dance, and Experienced Workers) and time (trial one to five) using Greenhouse-Geisser estimates where Mauchly’s test indicated that the assumption of sphericity had been violated. To ensure the assumptions of parametric statistics were met, pre hoc tests of normality were performed using Shapiro Wilk’s test and tests of homogeneity of variance were performed using Levene’s test for one-way ANOVA and Box’s test for two-way ANOVA. Post hoc tests for each ANOVA were corrected using Gabriel’s procedure to account for unequal sample sizes, and Games-Howell procedure where a violation of the assumption of equality of covariance was found.

To examine how performance changed as a function of the specificity of each group’s experience (from Inexperienced to Dance to Experienced Workers), linear trend analyses were performed for both the initial trial alone as well as across trials. The initial trial results for each CS variable were normally distributed and thus linear trend analyses were performed as follow up tests to the one-way ANOVAs. However, subsequent trials were occasionally not normally distributed (p < .05). While the repeated measures ANOVA is widely considered robust to violations of normality [21, 22], linear trend analyses were carried out using the non-parametric Jonckheere-Terpstra tests [23, 24].

Cohen’s *d* effect sizes [25] were calculated for each main effect and interaction to evaluate the magnitude of statistically significant differences. To examine how adaptation across trials differed between groups, effect sizes were calculated using the first and last trial for each group. Where homogeneity of variance was violated effect sizes were calculated using Glass’s delta [26]; otherwise, Hedge’s *g* effect sizes were calculated to account for unequal sample sizes [27]. For ease of interpretation, effect sizes are reported as ‘ES’.

All data reduction and analyses were performed using Matlab R2014a, MathWorks Inc., Natick, Massachusetts, USA). All statistical analyses were performed using IBM SPSS Statistics 20 (IBM Corporation, Armonk, NY, USA).

## Results

### Group differences in responses to the 1^st^ trial

The primary objective was to compare the differences in balance reactions during the first trial (novel trial) across the groups to determine if prior experience influenced the response to the initial exposure (S1 File).

#### Total time spent performing change-in-support strategies (%Trial)

**Fig 1:**
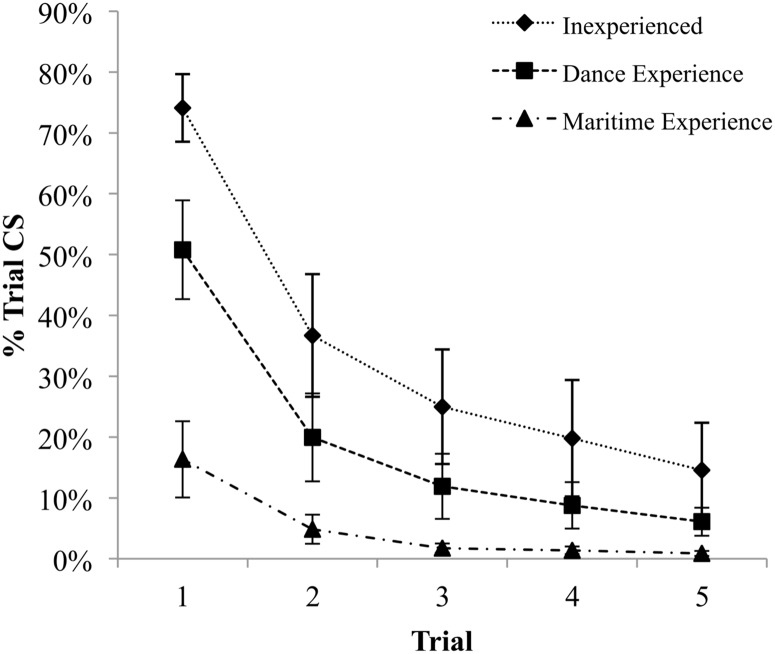
Percentage of trial spent performing change-in-support strategies. Data presented as group means with error bars representing standard error.

Statistically significant differences were found between groups for % Trial CS (*F*_(2, 36)_ = 18.44, *p* < .001, ES = 2.02). Post hoc testing revealed a greater % Trial CS in Inexperienced (Mean (*M*) = 70.10%, Standard Error (*SE*) = 5.56) compared to Experienced Workers (*M* = 16.35%, *SE* = 6.28) (*p* < .001, ES = 2.68), as well as greater % Trial CS in the Dance group (*M* = 50.80%, *SE* = 8.13) compared to Experienced Workers (*p* < .01, ES = 1.30). While post hoc testing did not reveal a statistically significant difference between the Inexperienced and Dance groups, a statistically significant linear trend was found across groups as a function of specificity of experience (*p* < .001, ES = 2.00), which can be observed in the first trial (Fig 1).

#### Total number of steps (#Steps)

**Fig 2:**
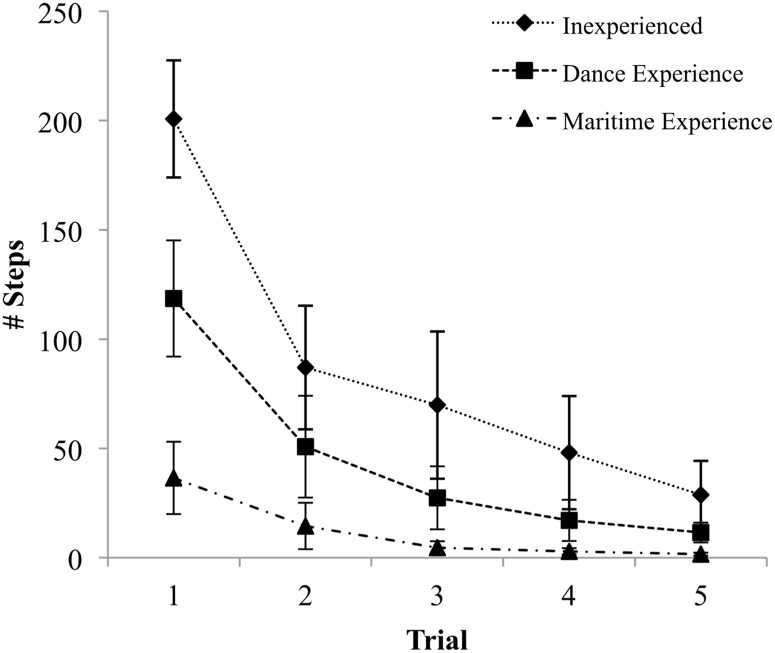
Total number of steps made per trial. Data presented as group means with error bars representing standard error.

Statistically significant differences were found between groups for # Steps (*F*_(2, 36)_ = 12.35, *p* < .001, ES = 1.66). Post hoc testing revealed a greater # Steps in Inexperienced (*M* = 200.75, *SE* = 26.78) compared to Experienced Workers (*M* = 36.50, *SE* = 16.53) (*p* < .001, ES = 2.12), as well as a greater # Steps in the Dance group (*M* = 118.62, *SE* = 26.58) compared to Experienced Workers and (*p* < .05, ES = 1.03). Despite post hoc testing not revealing a statistically significant difference between the Inexperienced and Dance groups, a statistically significant linear trend was found across groups as a function of specificity of experience (*p* < .001, ES = 1.65), which can be observed in the first trial (Fig 2).

#### Total number of multiple step responses (#Multistep)

**Fig 3:**
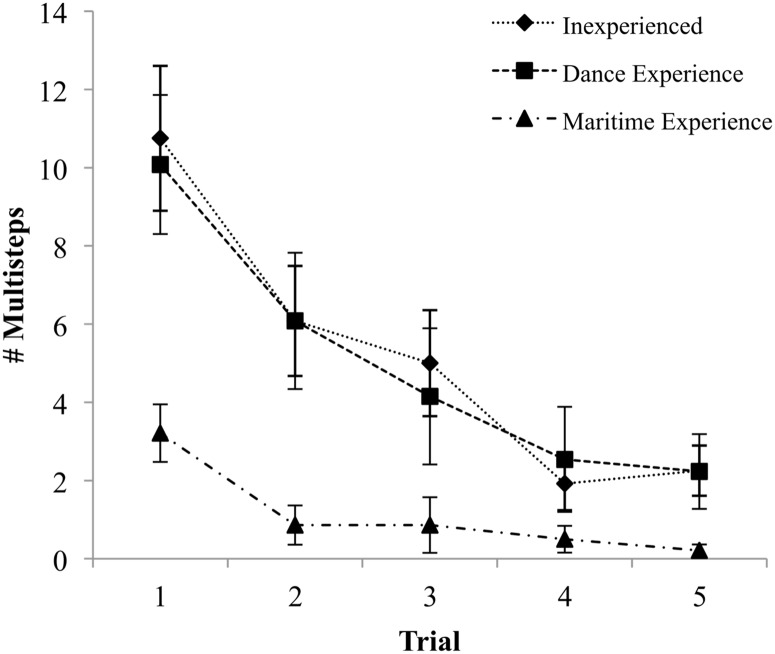
Number of multistep change-in-support reactions per trial. Data presented as group means with error bars representing standard error.

Statistically significant differences were found between groups for the number of multistep responses (*F*_(2, 36)_ = 8.03, *p* = .001, ES = 1.33). Post hoc testing revealed greater #Multisteps in Inexperienced (*M* = 10.75, *SE* = 1.86) compared to Experienced Workers (*M* = 3.21, *SE* = .74) (*p* < .01, ES = 1.57), as well as greater #Multisteps in the Dance group (*M* = 10.08, *SE* = 1.78) compared to Experienced Workers (*p* < .01, ES = 1.41). Statistically significant difference were not found between the Inexperienced and Dance groups; however, a statistically significant linear trend was found across groups as a function of specificity of experience (*p* < .01, ES = 1.19) which can be observed in the first trial (Fig 3).

### Group differences in short term adaptation (across trials)

The secondary objective was to compare the short-term adaptation (across all five trials) between groups as a reflection of the changes in central state with repeated exposure.

#### Total time spent performing change-in-support strategies (%Trial)

% Trial CS decreased significantly across trials for all groups (*F*_(1.89, 67.91)_ = 67.87, *p* < .001, ES = 2.74) with a significant interaction between trial and group indicating that groups learned at different rates (*F*_(3.77, 67.91)_ = 6.96, *p* < .001, ES = 1.24) (Fig 2). These different rates can be quantified by the effect size of the difference between the first and last trial of each group; Inexperienced participants demonstrated the greatest improvement (ES = 2.54), followed by the Dance group (ES = 2.08), and finally Experienced Workers (ES = .93).

% Trial CS was significantly different between groups (*F*_(2, 36)_ = 7.88, *p* < .01, ES = 1.33) (Fig 2). Post hoc testing revealed that Inexperienced participants demonstrated greater % Trial CS (*M* = 34.03%, *SE* = 5.36) than Experienced Workers (*M* = 5.04%, *SE* = 4.97) (*p* < .01, ES = 1.56). However, the differences between the Dance (*M* = 19.50%, *SE* = 5.15) and other groups did not reach statistical significance.

A statistically significant linear trend in performance was found on each trial (*p* < .001, ES = 1.50; *p* < .001, ES = .99; *p* < .001, ES = 1.14; *p* < .01, ES = .82; *p* < .005, ES = .86 for trials 1 to 5, respectively) indicating that groups spent less time changing support strategy as a function of the specificity of their balance experience, from Inexperienced to Dance to Experienced Workers (Fig 1).

#### Total number of steps (#Steps)

The # Steps decreased significantly across trials (*F*_(1.93, 69.33)_ = 38.15, *p* < .001, ES = 2.06) with a significant interaction between group and trial indicating that groups reduced their steps at different rates (*F*_(3.85, 69.33)_ = 5.19, *p* < .001, ES = 1.08) (Fig 3). These different rates can be quantified by the effect size of the difference between the first and last trial of each group; the Inexperienced group demonstrated the greatest improvement (ES = 2.27), followed by the Dance group (ES = 1.56), and finally Experienced Workers (ES = .80).

The number of steps used differed significantly between groups (*F*_(2, 36)_ = 6.14, *p* < .01, ES = 1.17) (Fig 3). Post hoc testing revealed that the Inexperienced group used significantly more steps (*M* = 86.9, *SE* = 15.69) than Experienced Workers (*M* = 12.03, *SE* = 14.52) (*p* < .05, ES = 1.38). However, the difference between the Dance group (*M* = 45.07, *SE* = 15.07) and Experienced Workers did not reach statistical significance, nor did the difference between the Dance and Inexperienced groups.

A statistically significant linear trend in performance was detected on each trial (*p* < .001, ES = 1.44; *p* < .001, ES = 1.03; *p* < .001, ES = 1.09; *p* < .01, ES = .75; *p* < .01, ES = .77 for trials 1 to 5, respectively) indicating that groups used fewer stepping strategies as a function of the specificity of their balance experience, from Inexperienced to Dance to Experienced Workers (Fig 2).

#### Total number of multiple step responses (# Multistep)

The number of CS strategy occurrences that resulted in multiple steps before a new support strategy was reached (# Multisteps) decreased significantly across trials for each group (*F*_(2.1, 75.42)_ = 18.6, *p* < .001, ES = 1.44). No significant interaction indicated that this decrease was similar across groups, which is reflected in the similar effect sizes representing the difference between the first and last trial for each group (Inexperienced ES = 1.32, Dance ES = 1.23, Experienced Workers ES = 1.09) (Fig 3).

The # Multisteps differed significantly between groups (*F*_(2, 36)_ = 11.61, *p* < .001, ES = 1.61) (Fig 3). Post hoc testing revealed that the Inexperienced group used significantly a greater number of multistep CS (*M* = 5.20, *SE* = .71) than Experienced Workers (*M* = 1.13, *SE* = .66) (*p* < .001, ES = 1.65). The Dance group also used a significantly greater number of multistep CS (*M* = 5.02, *SE* = .67) than Experienced Workers (*p* < .01, ES = 1.57). The difference between the Inexperienced and Dance groups did not reach statistical significance.

A statistically significant linear trend in performance was detected for the first three trials and the final trial (*p* < .005, ES = .97; *p* < .005, ES = .93; *p* < .005, ES = .93; *p* = .051, ES = .56; *p* < .01, ES = .77 for trials 1 to 5, respectively), indicating that for the first three and fifth trials groups used fewer multistep CS strategies as a function of the specificity of their balance experience, from Inexperienced to Dance to Experienced Workers (Fig 3).

## Discussion

One’s ability to use previous experiences to help successfully control balance is essential to fall prevention. In instances where a postural perturbation is truly novel (i.e. there is no previous experience to draw upon), the use of adaptations modelled on prior experiences would not be possible. In such situations individuals may use any previous balance experiences/training, regardless of their nature and similarity to the perturbation at hand, to assist in response control. While much is known regarding the influence of short term previous experiences on response development, the influence of long term prior experiences remains widely unknown. The question becomes, how do these prior experiences, no matter how remote or different, influence response development and execution? Does the central nervous system store precise perturbation properties for each situation or does it use more generalizable movement patterns that can then be adapted and fine-tuned for the situation using control parameters for tighter control once the perturbation is present? Results of this study suggest specificity of experiences plays an important role in central state setting of balance control.

Many studies have demonstrated that various types of motor training including slack line walking [28], recreational soccer [29], and dance [14, 15] improve balance during quiet standing. The extent to which these types of training transfer to novel balance tasks has received comparatively less attention from researchers. Chapman and colleagues [30] found limited improvements in dual task for trained surfers, while Asseman and colleagues [31] did not find transferability of expertise of hand standing to upright stance postural stability in elite gymnasts. In the current study, the optimization of the dancers was not as great as individuals with long-term training in wave motion environments. This suggests that response choice is highly dependent on past experiences and that specificity of these experiences to the current stimulus is necessary to achieve optimal results. Experienced maritime workers have developed a control set for the wave motions based on their past experiences on boats and therefore assume that the properties of the motions that they are about to experience will be similar to those despite never spending time on the specific simulator or being exposed to the wave motions used in this study. The dancers also appear to rely on their previous experiences, or skill, that focus heavily on balance training and control of the postural stabilizing muscles, to attempt to optimally control their centre of mass and resultant postural stability when exposed to the novel perturbation. They do not appear to be able to do this as effectively as the experienced workers however. Therefore, while some transferability in experiences is possible, optimal gains in central set require experiences closely associated to the novel perturbation while diverse enough to accommodate a wide range of potential perturbation characteristics. The transferability is likely associated with two different factors: 1) familiarity with stimulus; and 2) balance control skill/ability. The benefits of having prior experience with the task-specific characteristics of the stimulus (perturbation), that is specific familiarity with general stimulus/waveform properties, can provide an adaptive advantage [6, 16]. The second aspect that may influence transferability may be linked to the underlying skill/ability to recover balance under a range of external disturbances. It is possible that the slightly improved performance across trials observed in the dancers is linked to the latter while the specific benefits associated with maritime workers may have been more strongly linked to the former.

While the first motion trial was an indication of the each group’s ability to use long-term experiences to react to a novel perturbation, performance during the subsequent trials gives insight into the role that these long-term prior experiences may play a role in the ability of postural set adaptation. Previous research into the systematic evaluation of short-term adaptations to transient perturbations has found individuals tend to modify response magnitudes without effecting muscle onset latencies based on expectations created immediately prior to the response [32]. In this current study, this ability to quickly adapt is evident in each group’s ability to rapidly adapt after the first trial. Learning and habituation of responses, however, varied significantly between groups, further indicating the effect of long term previous learning on central set. While the Experienced Workers group did show evidence of adaptations across trials, as seen through reductions in time spent performing CSs, steps, multistep reactions and grasps, reductions were lower in magnitude compared to the dance and control groups. These adaptations were likely due to the fact that their responses reached an optimal plateau near the outset, therefore providing less room for further improvement. Results for the Inexperienced group closely resemble the rapid habituation observed in continuous low acceleration perturbing environments and high acceleration transient perturbations that show significant improvements and adaptations after the first trial [6, 7, 18]. The slightly faster adaptations of the dancers when compared to the Inexperienced group may be due to the fact that dancers have learned to rely on vision and enhanced somatosensory abilities and increased proprioceptive feedback that may strengthen synergist muscle activity and limb coordination in response development [33, 14].

Balance control is a sophisticated process that involves reactive and predictive control of multiple segments and the ability to move in multiple degrees of freedom at any time. In order to successfully meet the challenges in a remarkably quick manner to prevent falling, optimization of control is required [1, 2, 3, 34]. In most situations, postural perturbations are task- and environment-specific. The large amounts of information gained from this knowledge of task and environment greatly helps develop effective anticipatory gains in controls and optimization is possible. However, sometimes optimal amount of information about the perturbation is not available, leading to greater challenges to produce an effective response. Treating this novel situation like a completely new environment and relying solely on reactive control may not the best option because perturbation onset and required response to prevent falling is faster than intake and processing of available peripheral information [32]. Instead the central nervous system appears to use previous experiences in balance control to assist in its response development in such a way as to ensure an optimal response strategy is produced as quickly as possible. This is evident in the abilities for trained dancers with no prolonged experiences on any form of moving platform to develop more optimized control when compared to those with no balance training or experience working at sea, as indicated by the statistically significant linear trend analysis. Dancers are primarily trained to voluntarily move their bodies while maintaining upright stance in challenging dynamics situations. This training gives them a strong representation of balance and volitional balance control, as indicated by their high performance in many functional balance tests [12, 14, 15]. However, the current findings indicate this does not necessarily translate into equivalent success when exposed to external perturbations to their balance because their training does not primarily involve reactive balance control to unexpected perturbations, although exceptions may exist depending on individual training experiences and genre of dance. Neural control of volitional and reactive movement differs in some fundamental ways, including speed of reaction, use of anticipatory postural adjustments and their modulation in real time [35]. Volitional movements, like those of dance, are slower than reactive responses to external perturbations, and often have highly developed anticipatory postural adjustments that are mostly absent in reactive balance control. Furthermore, as a result of the speed of the response initiation, balance reactions must be completely modulated in “real time” as opposed to volitional movements that can be at least partially pre-planned [1, 35]. Due to these differences, training volitional movements does not necessarily result in effective training of reactive balance control [36].

Ambulatory people are continually subjected to threats to postural stability for which they must quickly develop and initiate effective responses to prevent a fall and potential injury. The variety of balance challenges that one may encounter in daily life makes it nearly impossible to train every potential outcome. While skill specific training is most beneficial, the results of this current study suggest that even previous experiences in seemingly unrelated balance activities such as dance, may still assist in central set development and the ability to effectively adapt to novel postural adaptations or learn a new skill that demands challenging dynamic stability. These findings are of clear practical importance for a number of reasons. Knowledge regarding an individual’s past experiences may be easily gathered and integrated into predictive screening tools for falls prevention. Furthermore, these results raise important considerations for the much-needed development of more effective training programs for fall prevention, which is essential to seniors, maritime workers, and people undergoing rehabilitation after injury or illness.

### Conclusions

Results of this study suggest that, in addition to short-term previous experiences, long-term previous experiences also have a significant influence on central set development and potential increases in neural gain prior to exposure to a novel postural perturbation. These results further highlight that diverse and seemingly unrelated previous experiences can influence the neural control of posture and balance in the development of compensatory responses.

## Supporting Information

S1 File Raw and summary stepping initation data for each participant. (XLSX)
